# Glial Patchwork: Oligodendrocyte Progenitor Cells and Astrocytes Blanket the Central Nervous System

**DOI:** 10.3389/fncel.2021.803057

**Published:** 2022-01-05

**Authors:** Heather M. Barber, Maria F. Ali, Sarah Kucenas

**Affiliations:** ^1^Program in Fundamental Neuroscience, University of Virginia, Charlottesville, VA, United States; ^2^Cell & Developmental Biology Graduate Program, University of Virginia, Charlottesville, VA, United States; ^3^Department of Biology, University of Virginia, Charlottesville, VA, United States

**Keywords:** glia, tiling, astrocyte, oligodendrocyte progenitor cell, development

## Abstract

Tiling is a developmental process where cell populations become evenly distributed throughout a tissue. In this review, we discuss the developmental cellular tiling behaviors of the two major glial populations in the central nervous system (CNS)—oligodendrocyte progenitor cells (OPCs) and astrocytes. First, we discuss OPC tiling in the spinal cord, which is comprised of the three cellular behaviors of migration, proliferation, and contact-mediated repulsion (CMR). These cellular behaviors occur simultaneously during OPC development and converge to produce the emergent behavior of tiling which results in OPCs being evenly dispersed and occupying non-overlapping domains throughout the CNS. We next discuss astrocyte tiling in the cortex and hippocampus, where astrocytes migrate, proliferate, then ultimately determine their exclusive domains by gradual removal of overlap rather than sustained CMR. This results in domains that slightly overlap, allowing for both exclusive control of “synaptic islands” and astrocyte-astrocyte communication. We finally discuss the similarities and differences in the tiling behaviors of these glial populations and what remains unknown regarding glial tiling and how perturbations to this process may impact injury and disease.

## Introduction

Tiling is a cellular behavior during development where, following specification, a population of cells migrate and become evenly dispersed, forming non-overlapping domains with their neighbors. There are many cell types in the central nervous system (CNS) that exhibit tiling behaviors including various neuronal and glial populations. For the purpose of this review, we define tiling as an emergent behavior driven by a number of different cellular processes. These processes include migration, proliferation, contact-mediated repulsion, and/or apoptosis. Different cell populations utilize some or all of these cellular processes during developmental tiling to ultimately achieve even dispersal and occupy distinct territories.

Neuronal tiling is a well-studied phenomenon and several known molecular mediators have been identified, studied, and are referenced here (Egea and Klein, 2007; Parrish et al., 2007; Hattori et al., 2008; Cameron and Rao, 2010; Grueber and Sagasti, 2010; Lefebvre, 2017). This body of work will not be discussed in this review, but we encourage those interested to access these references. Interestingly, although glial tiling is routinely described in the literature, there has been little investigation into the molecular mechanisms that contribute to the various cellular processes that drive this process (Bushong et al., 2002, 2004; Halassa et al., 2007; Hughes et al., 2013; De Biase et al., 2017). In this review, we discuss what is currently known about developmental tiling behaviors of two major glial populations: oligodendrocyte progenitor cells (OPCs) and astrocytes. First, we will describe the process of OPC tiling, which is regulated by the cellular behaviors of migration, proliferation, and contact-mediated repulsion (CMR). Next, we will discuss what is currently known about the different molecular mediators that influence each of these cellular processes and propose a model for how these cellular behaviors interact to produce the even distribution of OPCs throughout the CNS. We will then discuss what is understood about astrocyte tiling and highlight areas of similarity and difference with OPC tiling. Finally, we will discuss how perturbations to tiling may affect neural homeostasis and underlie disease. Ultimately, a better understanding of the cellular and molecular mechanisms that mediate glial tiling will lead to a fundamental understanding of how the nervous system develops under physiological conditions and could shed light on mechanisms that are perturbed after injury or in disease.

### Oligodendrocyte Progenitor Cell Tiling

**Figure 1:**
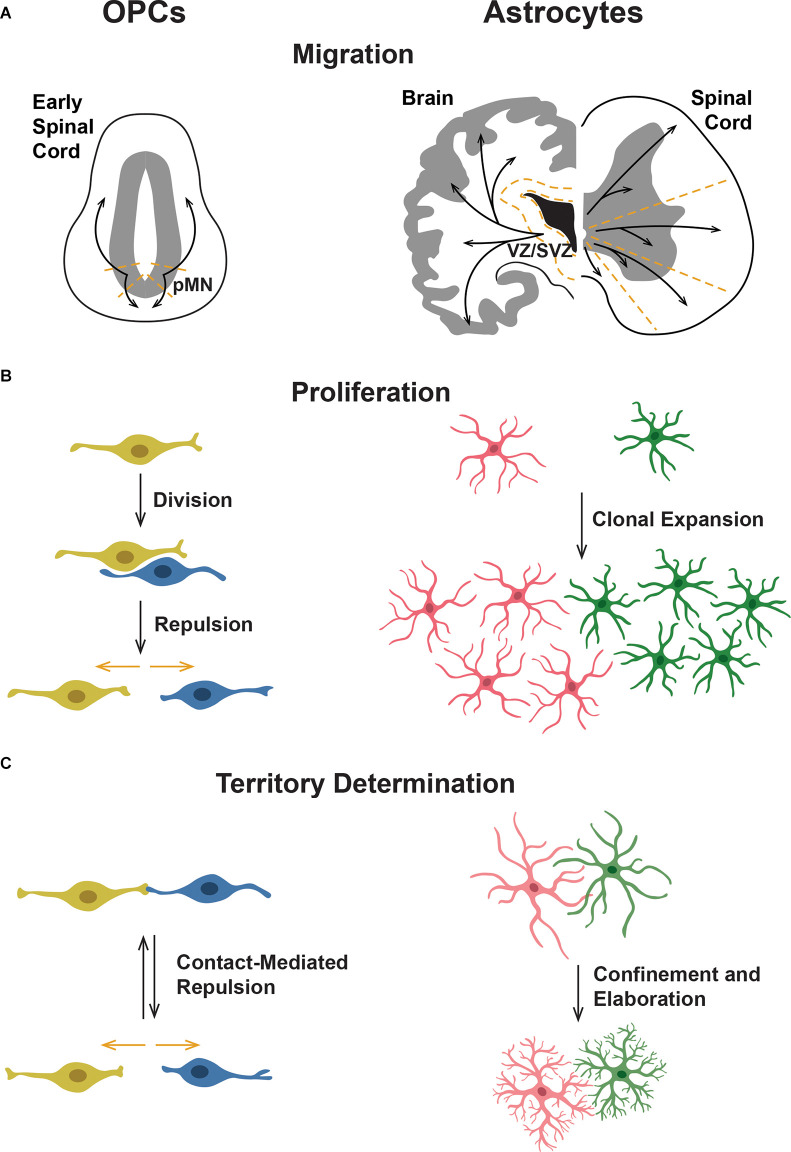
Comparison of oligodendrocyte progenitor cell (OPC) and astrocyte tiling behaviors. **(A)** OPCs migrate ventrally and dorsally from their origin in the ventral spinal cord motor neuron progenitor (pMN) domain. Astrocytes originate in the ventricular (VZ) and subventricular zones (SVZ) and migrate throughout the gray and white matter of the CNS. In the spinal cord, astrocytes migrate radially into separate domains. **(B)** OPCs divide and then are repelled from one another through CMR, leading to an equal spread across the neuropil. In astrocytes, pioneer astrocytes spread throughout the CNS and then clonally divide to cover the neuropil. **(C)** OPCs form their domains through CMR, during which contact between two OPCs leads to both OPCs retracting their processes and moving away from one another. This process occurs throughout the life of an OPC, leading to equilibrium. In contrast, immature astrocytes overlap extensively and then establish their domains over time. Astrocyte processes become more elaborately branched yet are more confined, suggesting some sort of retraction or pruning of processes.

During vertebrate development, OPCs are specified from gliogenic precursors in the CNS, which consists of the brain and spinal cord (Barres et al., 1993; Dawson et al., 2000). Most OPCs are specified from ventral precursor cells marked by expression of the basic helix-loop-helix transcription factor *Olig2*, which differentiate into *Sox10*-positive OPCs (Warf et al., 1991; Noll and Miller, 1993; Lu et al., 2000; Zhou et al., 2001; Park et al., 2002). Following the specification, OPCs rapidly disperse throughout the CNS until they occupy distinct, non-overlapping territories (Cai et al., 2005; Kirby et al., 2006; De Biase et al., 2017; Ravanelli et al., 2018). This process of OPC dispersal is termed developmental OPC tiling and is comprised of three main components: migration, proliferation, and CMR (Figures 1A–C; Kirby et al., 2006; Cameron and Rao, 2010; Hughes et al., 2013; Ali et al., 2021). In the literature, most investigations of OPC development focus on either OPC specification or differentiation into oligodendrocytes, the myelinating cells of the CNS (Richardson et al., 2006; Bergles and Richardson, 2015; Kearns et al., 2015; Crawford et al., 2016; Nishiyama et al., 2016; Chapman et al., 2018; Ravanelli et al., 2018; Hayashi and Suzuki, 2019; Kuhn et al., 2019; Perlman et al., 2020). These studies, while necessary for identifying markers of OPCs and investigating OPC development, maturation in myelin-producing cells, and response to injury, leave out a critical aspect of development where they interact and perform tiling behaviors that persist throughout their life in the CNS.

Based on current studies and a handful of identified mediators of OPC tiling behaviors, it is clear that a comprehensive understanding of the molecular mediators that guide OPC development is needed to understand how individual molecular mediators work together to influence individual OPCs that ultimately become evenly dispersed throughout the spinal cord. While we know that OPCs become evenly tiled throughout the brain, the majority of studies investigating developmental OPC tiling are conducted in the developing spinal cord. Therefore, we will focus on tiling behaviors of OPCs specified from ventral spinal cord motor neuron progenitor (pMN) domain precursors (Warf et al., 1991; Noll and Miller, 1993; Zhou et al., 2001; Park et al., 2007). In mammals, OPCs are also specified in the forebrain and utilize vasculature during initial migration, which is reviewed in Xia and Fancy (2021). Ventral precursors that give rise to OPCs are marked by their expression of the transcription factor *Olig2*, and subsequently, expression of *Sox10* beginning around 36 h post fertilization (hpf) in zebrafish, embryonic day 12.5 (E12.5) in mouse, and 10 weeks gestational age during human fetal development (Lu et al., 2000; Zhou et al., 2001; Ravanelli et al., 2018; van Tilborg et al., 2018). Based on *in vivo* studies, OPC tiling begins immediately following specification, with OPCs migrating out of the pMN domain both dorsally and ventrally (Figure 1A). From the moment they are specified, these cells exhibit CMR and use this behavior to control contact with neighboring OPCs and their own processes (Figure 1C; Kirby et al., 2006; Huang et al., 2020). Following their initial migration, OPCs begin to proliferate (Figure 1B). Newly born daughter cells exhibit CMR and migrate rapidly away from one another as they continue their migratory journey within the spinal cord (Kirby et al., 2006; Huang et al., 2020). As OPCs begin to occupy their ultimate territories, axon-OPC interactions also influence OPC proliferation (Jepson et al., 2012). As the population of OPCs throughout the spinal cord grows, OPCs become evenly distributed with CMR actively facilitating a consistent minimum distance between neighboring OPCs. Ultimately, OPCs reach a steady state of dispersal throughout the spinal cord that is maintained through the same developmental tiling behaviors of migration, proliferation, and CMR, as well as apoptosis in adult OPC tiling (Hughes et al., 2013; Birey et al., 2017).

Given this framework for how OPC tiling behaviors are related to each other during development, new investigations are needed that take a comprehensive approach to understand how the molecular mediators of each of these processes are related to each other and drive the emergent behavior of global tiling. Below, we will summarize what is known about the mediators that control these individual phenomena. We will then propose a model for how each of the developmental tiling behaviors interacts to achieve OPC dispersal.

### Chemotactic Signals That Influence Directional OPC Migration

The bulk of investigations into mediators of OPC migration has focused on the contributions of secreted chemoattractant and chemorepellent molecules within the developing spinal cord in promoting directional OPC migration (Sugimoto et al., 2001; Spassky et al., 2002; Tsai et al., 2002; Yan and Rivkees, 2002; Jarjour et al., 2003; Lalive et al., 2005; Tsai et al., 2006; Ohya et al., 2007; Frost et al., 2009; Mela and Goldman, 2013). The numerous signals identified as modulators of OPC migration are described as a molecular orchestra in a 2005 review and work done since then has continued to make this orchestra of chemotactic signals more complex (de Castro and Bribián, 2005).

**Figure 2:**
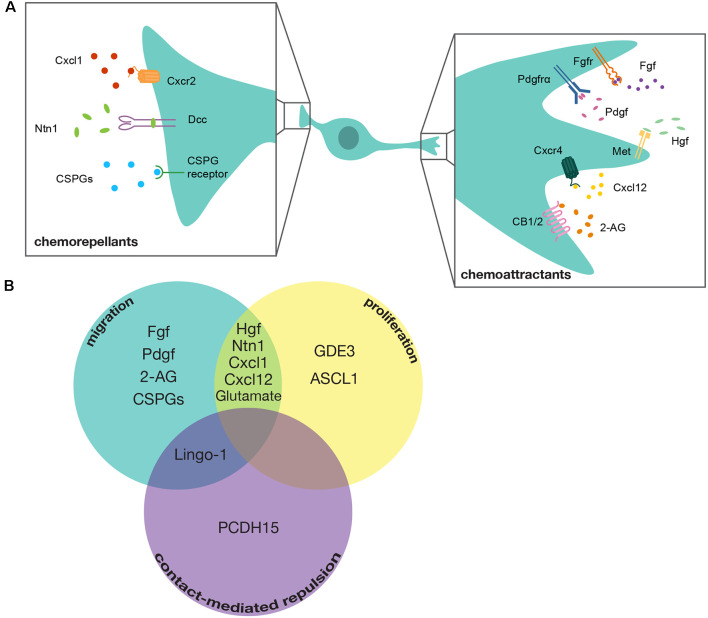
Mediators of OPC tiling. **(A)** Diagram of a developing OPC in the spinal cord with chemotactic signaling molecules that guide either chemorepellent or chemoattractant migration. Chemoattractants include: Fibroblast growth factor (Fgf) signaling through its receptor Fibroblast growth factor receptor (Fgfr), Platelet-derived growth factor (Pdgf) signaling through its receptor Platelet-derived growth factor receptor alpha (Pdgfrα), Hepatocyte growth factor (Hgf) signaling through its receptor Mesenchymal-epithelial transition (Met), C-x-c motif chemokine ligand 12 (Cxcl12) signaling through its receptor C-x-c chemokine receptor 4 (Cxcr4), and 2-Arachidonoylgylcerol (2-AG) signaling through its receptors Cannabinoid receptor type 1 and type 2 (CB1/2). Chemorepellents include: C-x-c motif chemokine ligand 1 (Cxcl1) signaling through its receptor C-x-c motif chemokine receptor 2 (Cxcr2), Netrin-1 (Ntn1) signaling through its receptor Deleted in colorectal carcinoma (Dcc), and Chondroitin Sulfate Proteoglycans (CSPGs) signaling through their cognate receptors. A cocktail of various CSPGs was used in this study, making it unclear exactly which CSPGs mediate this process. **(B)** Venn-Diagram demonstrating mediators that contribute to multiple tiling processes. GDE3, Glycerophosphodiester phosphodiesterase 3; ASCL1, Achaete-Scute Family BHLH Transcription Factor 1; Lingo1, Leucine-Rich Repeat and Immunoglobulin-like Domain Containing Nogo Receptor-Interacting Protein 1; PCDH15, Protocadherin Related 15.

Chemoattractant molecules that induce positive directional migration of ventral spinal cord-derived OPCs include Fibroblast Growth Factor (FGF), Platelet Derived Growth Factor (PDGF), Hepatocyte Growth Factor (HGF), Endocannabinoid 2-Arachidonoylglycerol (2-AG), and C-x-c motif Chemokine Ligand 12 (CXCL12; Yan and Rivkees, 2002; Ohya et al., 2007; Frost et al., 2009; Mela and Goldman, 2013; Sanchez-Rodriguez et al., 2018; Watson et al., 2020). Figure 2 provides a comprehensive overview of the signaling pathways, including ligands and receptors, reported to be involved in regulating chemotactic behaviors in OPCs. OPC migration is also controlled by chemorepellent molecules that either reverse or stop the directional migration of OPCs. Identified mediators include C-x-c motif Chemokine Ligand 1 (CXCL1), Netrin-1 (NTN1), and Chondroitin Sulfate Proteoglycans (CSPGs; Tsai et al., 2002; Jarjour et al., 2003; Tsai et al., 2006; Sun et al., 2017; Watson et al., 2020).

While each of these chemotactic molecules influences OPC migration, less is known about the downstream effectors that produce the changes to OPC migration. However, a handful of these studies demonstrated that the application of different chemokines to OPC cell cultures resulted in altered expression of cytoskeletal rearrangement proteins. For example, NTN1 signaling in OPCs increased RhoA activity resulting in increased process branching and migration away from the chemorepellent source (Rajasekharan et al., 2010). Similarly, CSPG4, also known as Neuron-Glial antigen 2 (NG-2), also regulates OPC migration by stimulating RhoA (Binamé et al., 2013). For a review of the molecules that influence OPC process outgrowth *via* the actin cytoskeleton, see Thomason et al. (2020). More work needs to be done to connect the various mediators that influence migration to downstream signaling pathways and how they affect OPC process outgrowth and cytoskeletal rearrangement.

These studies demonstrate that there are many chemotactic signals that influence OPC migration during development. However, the majority of studies that investigate mediators of OPC migration were conducted *in vitro*, which makes it difficult to discern exactly when these signaling pathways would be active during *in vivo* development, where the secreted mediators of these signaling cascades are being released, and how individual cells interpret these many cues for their navigation. For example, the role of Met signaling was extensively shown to influence OPC migration and proliferation *in vitro* (Yan and Rivkees, 2002; Lalive et al., 2005; Ohya et al., 2007). However, mouse mutants for *Met* are embryonic lethal. Recently, we utilized zebrafish as a vertebrate model and demonstrated that Met signaling is required for initial OPC migration (Ali et al., 2021). Utilizing both CRISPR/Cas9-mediated *met* mutagenesis and dominant negative Met transgenic fish, we demonstrated that loss of Met signaling significantly reduced the number of migrating OPCs during developmental tiling. However, a small population of OPCs was still able to migrate in the absence of Met signaling, demonstrating that a single cue is not responsible for OPC migration and that the OPC population is heterogeneous in its response to distinct cues (Ali et al., 2021). This study demonstrates the necessity of investigating glial dynamics *in vivo* and the importance of using multiple animal models.

Similarly, in the handful of *in vivo* studies that assess OPC migration in an altered chemotactic environment, particularly those that investigated the loss of NTN-1 signaling, there was often a population of OPCs that was still able to migrate (Tsai et al., 2002, 2006). This indicates that different populations of OPCs respond to different chemotactic signals or that in the absence of some cues, these cells are still capable of navigating their environment. In order to truly understand how OPCs sense their position in the nervous system and migrate, a comprehensive, *in vivo* approach to studying the combinatorial effects of these chemotactic signals is needed to parse out which populations of OPCs are sensitive to each single and combination of chemokines. An intriguing possibility is that these chemotactic responses of OPCs are regulated by levels of receptor expression. It is possible that different subsets of OPCs express different combinations of these receptors at different timepoints, or that they regulate receptor levels based on the environment they are in, and this differential expression can mediate local OPC dispersal that ultimately results in global distribution throughout the CNS. There are many new RNA-sequencing data sets that demonstrate differential expression of various chemokine receptors in OPCs from different regions of the CNS and at different developmental timepoints (Zhang et al., 2014, 2016; Chamling et al., 2021). A thorough examination of the different populations of OPCs identified in these RNA-sequencing investigations and the chemokine receptors expressed within each population could reveal the relative contributions of each chemotactic pathway to induce OPC migration during developmental tiling.

### Chemotactic Mediators of OPC Proliferation

Following initial migration, OPCs exhibit robust proliferation in both the dorsal and ventral spinal cord. Numerous investigations have explored various mediators of OPC proliferation in response to demyelinating events and spinal cord injury (Patel et al., 2012; Li et al., 2018; Ying et al., 2018; Adams et al., 2021). However, only a handful of studies have sought to identify mediators of developmental OPC proliferation. The majority of proposed mediators have been identified for their contribution as both chemotactic and mitogenic signals for OPCs during development (Figure 2). For example, CXCL1 and CXCL12 have opposite effects on OPC migration but are both purported to stimulate OPC proliferation (Watson et al., 2020). Additionally, the Met signaling pathway promotes OPC proliferation as well as migration (Ohya et al., 2007; Ali et al., 2021). The C-x-c motif ligands were investigated in the developing cortex, while the Met signaling pathway was investigated in the spinal cord, which indicates that regional differences in OPC populations could regulate which mitogens influence OPC proliferation during developmental tiling. These OPC tiling mediators that influence both migration and proliferation could be essential for ensuring the rapid expansion of the OPC population that must become evenly distributed to differentiate into myelinating oligodendrocytes.

Beyond these dual mediators of migration and proliferation, a handful of canonical mediators of proliferation are known, including Glycerophosphodiester phosphodiesterase 3 (GDE3), which negatively regulates OPC proliferation and Achaete-Scute Family BHLH Transcription Factor 1 (ASCL1), a transcriptional regulator that is required to stimulate OPC proliferation (Kelenis et al., 2018; Dobrowolski et al., 2020). The identification of these mediators of OPC proliferation indicates that achieving the appropriate number of OPCs during development involves a complex balance of positive and negative modulators. Additionally, a recent study demonstrated that axon-OPC interactions also play a role in regulating OPC proliferation and that increased Ca^2+^ signaling through α-amino-3-hydroxy-5-methyl-4-isoxazolepropionic acid (AMPA) receptors at axon-OPC synapses directly increases OPC proliferation (Chen et al., 2018). This feedback loop from neuronal signaling to increasing OPC proliferation indicates that the niche that OPCs occupy also directly regulates the number of OPCs present. However, recent work from our lab, which mutated the AMPA-receptor subunit Glutamate Receptor 4A (GluR4A), demonstrated no change in the number of OPCs in the spinal cord (Piller et al., 2021). These results indicate that more work needs to be done to identify which AMPA receptors influence OPC proliferation. Taken together, these studies demonstrate that initial OPC proliferation is regulated through intrinsic mediators during the migratory phase, but proliferation is later regulated by axon-glial signaling in the OPC niche following developmental migration.

### Proposed Mediators of OPC CMR

The least investigated but possibly most intriguing process of OPC tiling is CMR. Foundational studies demonstrate that migratory OPCs retract their process and alter their direction of migration following direct contact with neighboring OPCs during development, after injury, and during adult OPC homeostasis to maintain a consistent minimum distance between and ensure the maintenance of non-overlapping territories (Kirby et al., 2006; Hughes et al., 2013). However, we know virtually nothing about the molecular mediators that govern this behavior, or if the same mechanisms are used under different physiological conditions. Because the process of CMR involves other cellular behaviors, including directed-migration and membrane process remodeling, it can be difficult to determine if a given mediator regulates CMR or if there is some other defect in the other cellular processes involved in successful repulsion.

To date, the majority of investigations into CMR have examined the role of this behavior in the dispersal of various neuronal cell types during development (Noren and Pasquale, 2004; Egea and Klein, 2007; Grueber and Sagasti, 2010; Villar-Cerviño et al., 2013). Canonical mediators of CMR are transmembrane proteins capable of bi-directional signaling, such as Eph-Ephrin signaling, Down Syndrome Cell Adhesion Molecules (DSCAMs), and Leucine-Rich Repeat And Immunoglobulin-Like Domain-Containing Nogo Receptor-Interacting Protein 1 (LINGO1; Zimmer et al., 2003; Noren and Pasquale, 2004; Millard et al., 2007; Mayor and Carmona-Fontaine, 2010; Figure 2). Interestingly, Ephrin signaling and LINGO1 have been implicated in influencing axon-OPC interactions and OPC positioning, which suggests that OPCs may be capable of utilizing these canonical CMR mediators of heterotypic interactions and, therefore, might also utilize these them in OPC-OPC interactions (Prestoz et al., 2004; Jepson et al., 2012). Additionally, a recent paper that conducted single-cell RNA-sequencing on OPCs derived from the human cortex found that *DSCAM* is uniquely enriched in these cells (Huang et al., 2020). However, knock-down of *DSCAM* using shRNA showed no effect on OPC CMR (Huang et al., 2020). Intriguingly, however, Huang et al. (2020), also found *PCDH15* (*protocadherin related 15)* is uniquely expressed by OPCs and demonstrated that inhibiting PCDH15 resulted in a failure of OPCs to separate and migrate away from each other following cell division. This exciting new discovery lays the groundwork for investigating the contribution of CMR in OPC tiling, and future discoveries of new mediators of this process are sure to increase the complexity of this essential component of OPC spacing.

### OPC Tiling Homeostasis and Injury Response

Developmental OPC tiling establishes the distribution of OPCs into distinct, non-overlapping domains throughout the CNS (Kirby et al., 2006). Following the completion of developmental tiling, a large population of OPCs differentiate into myelinating oligodendrocytes, while the remaining OPCs remain undifferentiated as adult OPCs, making up approximately 10% of the total cell population of the CNS (McTigue and Tripathi, 2008; Bergles and Richardson, 2015; Hayashi and Suzuki, 2019). Tiling and homeostasis of adult OPCs are maintained through the same developmental tiling behaviors of migration, proliferation, and CMR (Hughes et al., 2013; Birey et al., 2017). However, adult OPCs also utilize apoptosis to maintain appropriate numbers (McTigue and Tripathi, 2008; Hughes et al., 2013). Adult OPC tiling, like developmental OPC tiling, is well-described phenomenologically, however, even less is known about how adult OPC behaviors are regulated (Hughes et al., 2013; Birey and Aguirre, 2015). One possibility is that OPCs utilize similar mechanisms in adult tiling as developmental tiling. For example, NTN1 is upregulated following OPC depletion and is required for OPCs to repopulate the cortex (Birey and Aguirre, 2015). Additionally, Met signaling, while downregulated in OPCs as they mature into oligodendrocytes, is upregulated in OPCs in the mouse models of multiple sclerosis (Lalive et al., 2005; Mela and Goldman, 2013). This work demonstrating that OPCs upregulate developmental OPC tiling mediators in response to injury and disease lays a foundation for further investigation into regulating OPC injury response by modulating developmental mediators OPC tiling.

In conclusion, OPC tiling is a complex process comprised of multiple cellular behaviors each of which is influenced by a number of different molecular mediators. In this review, we discussed known molecular mediators that regulate the developing OPC tiling processes of migration, proliferation, and CMR. Each of the mediators discussed influences one or more of these behaviors. However, the exact timing of expression within OPCs and how these mediators and cellular behaviors of tiling are influencing each other is still unclear. Future work that investigates multiple mediators of OPC tiling and their relation to one another will be critical to developing a comprehensive understanding of OPC tiling and necessary to facilitate the comparison of OPC tiling to the tiling of other glial populations.

## Introduction to Astrocyte Tiling

OPCs are not the only glial population that exhibits tiling behavior. Astrocytes, which make up between 20 and 40% of the total brain cell count (Khakh and Sofroniew, 2015), cover the entire CNS with minimal overlap (Bushong et al., 2002). Previously thought to be largely passive, astrocytes are now known to have a myriad of essential functions such as blood brain barrier mediation, synaptic regulation, axon guidance, and more (Araque et al., 1999; Powell and Geller, 1999; Gordon et al., 2007; Sofroniew and Vinters, 2010; reviewed in Sloan and Barres, 2014). Arguably, their most well-known function is their participation in tripartite synapses. Astrocytes contact synapses with processes known as perisynaptic astrocyte processes (PAPs) to form tripartite synapses, where they contribute to synapse efficiency through buffering and promote either stability or pruning of the synapse as necessary (Araque et al., 1999; Oliet et al., 2001; Blanco-Suárez et al., 2017). A single astrocyte can contact over 100,000 synapses in rats and over 2 million synapses in humans (Bushong et al., 2002; Oberheim et al., 2009). This information has led many to suspect that astrocytes play an even more important role in brain function and neuronal communication than is currently understood.

Originally named for their “star shaped” appearance (Lenhossék, 1893), astrocytes actually have vastly different morphologies depending on their position and function within the CNS (Khakh and Sofroniew, 2015; Chai et al., 2017; Zhou et al., 2019). In rodents, the major categories of astrocytes are protoplasmic and fibrous. Protoplasmic astrocytes are the most abundant glial cell in the gray matter and are defined by their bushy, sponge-like morphology and low Glial Fibrillary Acidic Protein (GFAP) expression (Bushong et al., 2004; Molofsky and Deneen, 2015). Fibrous astrocytes are found in the white matter and have thicker, less ramified processes and high GFAP expression (Molofsky and Deneen, 2015). There exist descriptions of other major subtypes in primates (Oberheim et al., 2009), as well as evidence for possible subtypes and specializations within and beyond these major classes (Bailey and Shipley, 1993; Hochstim et al., 2008; Zhou et al., 2019; Batiuk et al., 2020). Protoplasmic astrocytes, in particular, occupy distinct individual areas with little overlap despite their extensive branching (Bushong et al., 2004), a phenomenon similar to the tiling behavior seen in OPCs and neurons. We refer to these areas controlled by a single astrocyte as “domains” or “territories”. This review will primarily focus on these protoplasmic astrocytes in the cortex and hippocampus, where there is extensive evidence for tiling.

### Evidence for Astrocyte Tiling

In one of the earliest descriptions of astrocyte tiling, astrocytes in the hippocampus were filled with dye to label fine processes and subsequently imaged (Bushong et al., 2002; Ogata and Kosaka, 2002). In contrast to earlier assumptions that astrocytes covered generally spherical domains with extensive overlap (Rohlmann and Wolff, 1996), Bushong et al. (2002) demonstrated that rat astrocytes inhabit largely exclusive domains which are not necessarily radially arranged around the soma. The shape of the domains occupied by the astrocytic processes varies, as does the position of the soma itself, allowing the astrocytes to fill the neuropil with minimal overlap, controlling volumes which fit together like puzzle pieces. Ogata and Kosaka (2002) further described the lack of process protrusion into the innermost areas of neighboring astrocytes in mice, in which the extent of overlap between two astrocytes is between 2 and 5% of one astrocyte’s total domain volume (Wilhelmsson et al., 2006). These observations imply that astrocyte morphology is somehow affected by neighboring astrocytes. Though domain shape varies, individual protoplasmic astrocytes in the mouse cortex occupy territories of similar volume (Halassa et al., 2007). There is also evidence that astrocyte densities and the volume controlled by a single astrocyte vary by brain region (Chai et al., 2017). This could be due to a variety of reasons, including the types of neurons in each region or available nutrients. These regional differences may also suggest more subtypes of astrocytes, or perhaps environmental cues informing the extent of growth and infiltration of populations of astrocytes based on the immediate environment and the needs of the other resident cell populations in the region. However, how astrocytes ultimately occupy their precise domains is unknown.

Several subsequent articles have confirmed the existence of tiling by protoplasmic astrocytes in the mouse, primate, and human cortices (Wilhelmsson et al., 2006; Halassa et al., 2007; Oberheim et al., 2008, 2009). Human astrocytes were larger and more complexly ramified in these studies (Oberheim et al., 2009). However, the extent of overlap remains relatively minimal (Oberheim et al., 2006, 2009). In addition to the studies in mammals, there is recent evidence that zebrafish astrocytes inhabit distinct domains by 6 days post fertilization (Chen et al., 2020), and that astrocyte-like cells in *Drosophila* also exhibit tiling (Stork et al., 2014). This demonstrates that tiling is not only a mammalian, or even only a vertebrate, behavior.

### Diversity of Astrocyte Tiling Behaviors

Although astrocytes typically do not have significant overlap with their neighbors, other glial cell types are able to freely infiltrate their territories and form overlapping domains, demonstrating that tiling is controlled differently when considering homo vs. heterotypic interactions (Bushong et al., 2002). This is even true for different astrocyte subtypes. Therefore, tiling is not a feature of all astrocytes and the extent varies by subtype. Human fibrous astrocytes interdigitate extensively in the white matter (Oberheim et al., 2009). Varicose projection astrocytes, which have only been found in higher order primates, extend their long processes into the domains of neighboring protoplasmic astrocytes (Oberheim et al., 2009), indicating that different subtypes do not necessarily tile with each other. It is unknown whether varicose projection astrocytes themselves tile, though they are sparsely distributed and therefore may only rarely interact. In general, astrocyte tiling appears to mainly be a feature of protoplasmic astrocytes. It is notable that there are molecular and functional differences between protoplasmic and fibrous astrocytes (e.g., protoplasmic astrocytes contact synapses while fibrous astrocytes contact nodes of Ranvier) which may help explain the difference in tiling behaviors (Sofroniew and Vinters, 2010).

While the overlap between astrocytes remains relatively small, overlap area is over 17 times greater in humans than in rodents, significantly more than the predicted 6.5 times increase due to the disparity in overall astrocyte volume alone (Oberheim et al., 2006, 2009). Why this occurs is unknown, though it is possible that the sheer number of synapses human astrocytes are in contact with means that more connections between astrocytes are necessary for them to function efficiently, which may lead to increased domain overlap.

In contrast to evidence in other mammals, studies of the ferret cortex have revealed an extensive overlap of astrocyte domains, up to 50% even into maturity, which is not explained by an increase in cell volume alone (López-Hidalgo et al., 2016). However, a morphologically distinct subtype consisting of pairs of astrocytes whose cell bodies touch, dubbed “kissing astrocytes,” showed less domain overlap compared to other astrocytes (López-Hidalgo et al., 2016). This behavior may be analogous to protoplasmic astrocyte behavior, though kissing astrocytes are less common. Therefore, even though tiling has been observed across multiple species, widespread protoplasmic astrocyte tiling is not necessary for brain function in mammals.

### Astrocyte Networks

**Figure 3:**
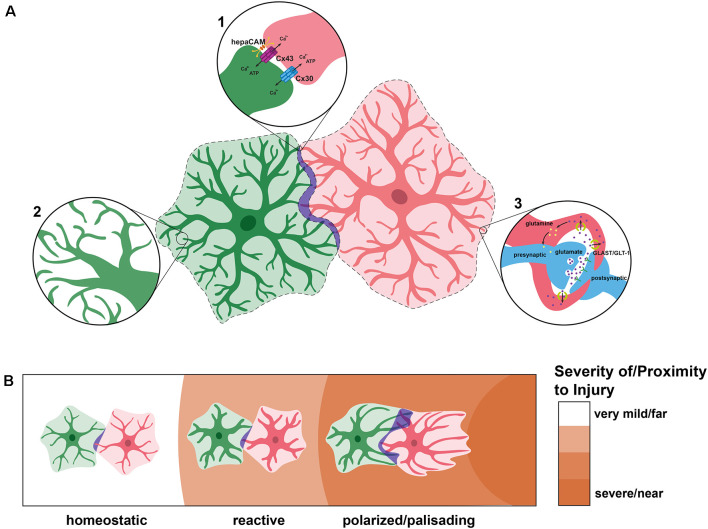
Astrocyte tiling under physiological and pathological conditions. **(A)** Under physiological conditions, individual astrocytes overlap very minimally (purple), creating defined domains (dashed lines). In these spaces of overlap, astrocytes connect with one another *via* connexins 30 (Cx30) and 43 (Cx43), the latter of which is localized through HepaCAM (inset 1). Within their domains, astrocytes become highly branched and elaborate, sometimes forming connections with their own processes (inset 2). This extensive branching is represented by the lighter overlay around each astrocyte. Astrocytes regulate synapses by wrapping around them and insulating them, taking up excess neurotransmitters such as glutamate, and recycling them (inset 3). Each astrocyte is able to connect with thousands of synapses, a feature which has increased interest in astrocyte domains and networks. **(B)** After injury or insult, astrocytes can become reactive and undergo morphological changes including thicker processes but do not generally send processes outside of their domains. With nearby or severe insult, reactive astrocytes generate polarized astrocytes which can become palisading. These astrocytes may extend processes towards the injury which can protrude into the domains of neighboring astrocytes.

Under physiological conditions, there is a small amount of overlap between astrocytic domains, somewhere between 2 and 5% (Ogata and Kosaka, 2002; Wilhelmsson et al., 2006). This minimal overlap may be explained by the fact that astrocytes contact each other through gap junctions (Dermietzel et al., 1989; Giaume et al., 2010; Mayorquin et al., 2018). This is a notable difference between OPCs and astrocytes. OPCs do not maintain contact with each other, as any contact leads to CMR, and there is thus no sustained overlap between OPC domains (Figure 1C). Astrocytes form networks in which individual astrocytes are connected to each other *via* connexins, particularly Cx43 and Cx30 (Dermietzel et al., 1989, 1991; Nagy et al., 1999; Giaume et al., 2010; Mayorquin et al., 2018; Figure 3A). Different processes of a single astrocyte can also be connected *via* Cx43 in a “reflexive” network (Giaume et al., 2010; Haseleu et al., 2013). This may be so that astrocytes can very quickly communicate to coordinate synapses within their own domains. However, reflexive gap junctions may primarily be used for regulating membrane and cytoskeletal organization (Wolff et al., 1998). Astrocyte networks can vary in size and shape and are selective, meaning that an astrocyte may exist within the same area as astrocytes connected into a network and not be connected to the network itself (Giaume et al., 2010). Astrocytes may use these networks to coordinate neuronal activity, making their selectivity and relationship to astrocyte domains even more intriguing. However, the extent to which the connections between astrocytes affect tiling is currently unknown, though Cx43 is important for astrocyte morphology, which could be an important contributor to tiling (Baldwin et al., 2021).

### Overview of Astrocyte Development

To begin to understand how astrocytes might achieve tiling, we will give an overview of the processes leading up to and including tiling. Like OPCs, in order to form exclusive domains which together cover the neuropil, astrocytes must migrate to their final destinations, proliferate, and choose their territories.

#### Astrocyte Migration and Proliferation

Much of the following will summarize information that is more extensively reviewed in Tabata (2015) and Schiweck et al. (2018). Cerebral cortex astrocytes originate from multiple parts of the brain, namely the ventricular zone (VZ), ventral forebrain, and subventricular zone (SVZ; Figure 1B). Radial glia (RG) in the VZ and ventral forebrain act as both scaffolds and neural stem cells that produce astrocytes (Tabata, 2015). Early in development, RG asymmetrically divide to create neurons and then switch to creating glial progenitor cells, some of which eventually become astrocytes (Tabata, 2015; Schiweck et al., 2018). RG later terminally differentiate into astrocyte precursors, specifically. These astrocyte precursors then migrate into cortical gray or white matter and further differentiate into protoplasmic or fibrous astrocytes, respectively (Tabata, 2015). Glial progenitors also migrate from the SVZ to become astrocytes (Tabata, 2015). Inhibiting proliferation in the SVZ reduces the number of protoplasmic astrocytes but not fibrous astrocytes, indicating that different astrocyte subtypes may come from different precursor populations (Tabata, 2015). It is currently unknown if the multiple origins for the same major astrocyte subtype are indicative of further specification within the subtype, though there is some evidence suggesting this may be so. Tsai et al. found that astrocyte progenitors migrate to specific regions based on their origin in the VZ and do not exhibit secondary migration (Tsai et al., 2012). Additionally, this study demonstrated that spinal cord astrocytes from one progenitor region will not infiltrate and rescue neighboring regions that have been depleted of astrocytes (Tsai et al., 2012), further indicating that these regions are somehow distinct from one another (Figure 1B). It is not yet clear whether these populations are distinct due to their separate origins, final locations, or a combination of both. Once progenitors reach their final destination, they continue to proliferate (Figure 1C). The majority of the brain’s protoplasmic astrocytes are produced locally by proliferating protoplasmic astrocytes (Tabata, 2015). While PAPs continue to remodel under physiological conditions (Blanco-Suárez et al., 2017; Schiweck et al., 2018), mature astrocytes are mostly quiescent and do not migrate further (Zhan et al., 2017). This stability is unlike OPCs, which are constantly remodeling processes and domains *via* CMR, leading to a distinct tiling process.

#### Astrocyte Territory Determination

Once astrocytes have migrated to and populated an area, they must somehow compartmentalize this area into separate, individual domains. However, little is known about how this is achieved and if it is governed by intrinsic, extrinsic, and/or environmental mechanisms. Most of what has been described focuses on the progression of astrocyte morphology throughout early development. As astrocytes develop, astrocytic processes branch more and become less “stringy” and more “spongiform,” a process sometimes described as “ramification” (Bushong et al., 2004; Chen et al., 2020). During ramification, processes also become more contained, extending less distantly from the cell body, and ultimately become confined to an astrocyte’s emerging individual territory (Bushong et al., 2004). Early in development (P7 in rats), astrocytes overlap extensively and establish their exclusive domains over time (established by P21; Bushong et al., 2004; Figure 1C). This implies that, unlike OPCs, astrocytes do not establish domains *via* CMR between membranous processes. Instead, their processes are overproduced, or “exuberant,” and later pruned and/or retracted. To our knowledge, the mechanisms behind the removal of branches and the determination of which branches are removed have not been investigated.

Recently, a new study described the astrocyte-enriched Hepatocyte Cell Adhesion Molecule HepaCAM (or GlialCAM) in astrocyte development and tiling. Using loss-of-function studies, this work demonstrated that *Hepacam* mutants had smaller territory volume during development, both *in vitro* and *in vivo* (Baldwin et al., 2021). Mosaic *Hepacam* conditional knockouts showed that wildtype astrocytes overlapped significantly more with knockout astrocytes than with other wildtype astrocytes, suggesting that HepaCAM controls territory competition (Baldwin et al., 2021). Interestingly, this study demonstrated that HepaCAM affects the localization and stabilization of Cx43, which is important for proper astrocyte morphology independent of its channel activity (Baldwin et al., 2021). Considering the relationships between Cx43, HepaCAM, and astrocyte morphology, it is possible that morphology is a key factor in how astrocytes determine appropriate overlap (Figure 3A). Future work is needed to determine the method by which Cx43 regulates astrocyte morphology and how this may relate to the gap junctions it forms between astrocytes.

## Astrocyte Tiling in Reactivity and Disease

The term “reactive” is used to describe astrocytes which have adopted distinct morphological and/or molecular phenotypes in response to a perturbation such as an injury or disease, with “reactive astrogliosis” referring to the process itself (Escartin et al., 2021). This definition covers a broad range of phenotypes. The effects of reactivity on an astrocyte depend on the type, severity, and duration of insult as well as the distance of the insult from the astrocyte (Schiweck et al., 2018). A more general discussion of the current knowledge and consensus on reactivity can be found in the review by Escartin et al. (2021). Here we will discuss the relationship between reactivity and tiling.

Wilhelmsson et al. (2006) investigated the changes in reactive astrocyte morphology after a cortical lesion to elucidate the effect of reactivity on astrocytic domains. In this work, the authors found that in comparison to nonreactive astrocytes, reactive astrocytes exhibit more main processes, thicker main processes, and a higher proportion of processes above a certain length. Intriguingly, despite these morphological changes, the processes still did not extend out of the astrocyte’s individual domain. Additionally, while astrocyte volume increased, the volume each astrocyte encompassed did not (Figure 3B). This is especially interesting given that, despite their similarities to immature astrocytes (e.g., re-entry into the cell cycle and increased proliferative ability), reactive astrocytes do not return to the increased interdigitation observed in immature astrocytes during development. Therefore, high levels of overlap during development are likely an important component of tiling under physiological, but not pathological, conditions. Similarly, reactivity without domain disruption has been observed in mouse models of Alzheimer’s disease (Oberheim et al., 2008). Therefore, domain disruption is not a hallmark of all reactive astrogliosis, and reactive astrocytes generally maintain minimal overlap between their domains.

In cases of more severe and/or long-lasting insults, astrocytes experience further changes in morphology and behavior. Subsets of astrocytes near insults can become “polarized”, with their processes elongating and orienting towards the damage (Bardehle et al., 2013; Figure 3B). Most polarized astrocytes are generated by mature, non-polarized astrocytes near the injury, which temporarily dedifferentiate and begin proliferating (Bardehle et al., 2013; Schiweck et al., 2018). The amount of proliferation by each de-differentiated astrocyte is low, approximately two daughter cells each (Bardehle et al., 2013). This polarization becomes more pronounced nearer to the injury until the astrocytes are considered “palisading” (Schiweck et al., 2018). When polarized and palisading astrocytes send out processes towards an injury site, these processes can infiltrate the domains of adjacent astrocytes (Schiweck et al., 2018; Figure 3B). However, these polarized astrocytes do not migrate (Bardehle et al., 2013). Instead, astrocytes near injury sites become permanently reactive in order to form a border, or “glial scar,” between the injury and healthy tissue, preventing necrotic spread (Schiweck et al., 2018). As there exist subtypes of astrocytes which do not tile, it is possible that these polarized and palisading astrocytes are a separate astrocyte subtype which do not exhibit the same tiling mechanisms as their progenitors.

While astrocytes forming glial scars can exhibit some domain disruption, most milder forms of insult do not invoke disruption, with the notable exception of epileptic seizures. Oberheim et al. (2008) induced seizures in three different models of epilepsy—injury-induced, drug-induced, and genetically susceptible—and observed the effects on astrocyte domains. All three models showed a significant increase in protoplasmic astrocyte overlap, the extent of which varied by model, without the characteristic polarization of astrocytes seen in severe reactive astrogliosis. Treatment with seizure medication led to a reduction in overlap, though reactive astrocytes were still observed in the injury site. This indicates that the loss of domain organization is related to the seizures themselves rather than the injury and subsequent reactive astrogliosis and that there are mechanisms involved in maintaining domains that can be turned off and back on in response to pathology. Therefore, seizure models may be an important tool in the understanding of astrocyte tiling.

There are some other potential tiling defect models. Astrocyte territory size is affected in models of other diseases such as Huntington’s disease (Zhou et al., 2019), though it is unclear if this affects tiling mechanisms or if it is only a morphological defect. It has also been proposed that human varicose projection astrocytes, which send projections into the territories of other astrocytes, may not be their own subtype but instead are astrocytes in a certain unknown state, either developmental or pathological (Falcone et al., 2022). Unfortunately, there is little information on the effects of disease on tiling itself or if perturbations to tiling may underlie the etiology of nervous system disorders.

## Remaining Questions and Future Directions

While the existence of astrocyte tiling is evident, the molecular mechanisms and processes behind these events remain largely unknown. In addition, the effects of many diseases and disorders on tiling are not often considered, and thus not explored. Therefore, there are possibly more neurological conditions which disrupt tiling or are caused and/or exacerbated by a loss of tiling but have not yet been shown to do so. The functional role of tiling is unknown, in part due to the lack of models in which it is disrupted, particularly without some other pathology involved. More studies comparing the ferret cortex with models that do exhibit tiling may be useful to determine how important tiling is to efficient astrocyte function. In addition, there is little information available on astrocyte tiling outside of the cortex and hippocampus, so it is unclear if the same tiling strategies described here are true for all astrocytes.

How astrocytes choose, compete for, and maintain their domains has not yet been well defined. While more astrocyte subtypes continue to be theorized and characterized, the effects of these categories on tiling and other astrocyte-astrocyte interactions should be considered in addition to those on astrocyte-neuron interactions. More attention is being given to the possibility that astrocytes coordinate the neurons within their domain. It follows that more emphasis may be placed on tiling and astrocyte networks in the future.

## Concluding Remarks

Astrocytes and OPCs display similar but discrete behaviors which we have referred to collectively as “tiling” (Figure 3). While OPCs utilize CMR to establish their domains, leading to dynamic non-overlapping territories, astrocytes employ a different method of gradual retraction or pruning of processes which results in the minimal but existing overlap. Astrocytes may need this overlap to establish communication networks *via* membrane contact, while OPCs are able to function properly without continuous contact. In the end, OPC tiling evokes the image of a tiled floor with separate pieces which do not touch and do not overlap. Meanwhile, astrocyte tiling is more reminiscent of a tiled quilt, in which there are separate pieces stitched together, touching and overlapping at their seams. While these behaviors look similar and it is useful to group them, the processes behind their construction and the functions they serve are distinct.

## Author Contributions

HB, MA, and SK: conceptualization, writing, and funding acquisition.

## Conflict of Interest

The authors declare that the research was conducted in the absence of any commercial or financial relationships that could be construed as a potential conflict of interest.

## Publisher’s Note

All claims expressed in this article are solely those of the authors and do not necessarily represent those of their affiliated organizations, or those of the publisher, the editors and the reviewers. Any product that may be evaluated in this article, or claim that may be made by its manufacturer, is not guaranteed or endorsed by the publisher.
